# Lipid Metabolism and Statin Therapy in Neurodegenerative Diseases: An Endocrine View

**DOI:** 10.3390/metabo15040282

**Published:** 2025-04-18

**Authors:** Antonella Di Sarno, Fiammetta Romano, Rossana Arianna, Domenico Serpico, Mariarosaria Lavorgna, Silvia Savastano, Annamaria Colao, Carolina Di Somma

**Affiliations:** 1 Section of Endocrinology, Endocrinology Diabetology and Andrology Unit, Department of Clinical Medicine and Surgery, University of Naples Federico II, Via Sergio Pansini 5, 80138 Naples, Italy; antonella.disarno@unina.it (A.D.S.); rossana.arianna@unina.it (R.A.); domenicoserpico@libero.it (D.S.); mariarosaria.lavorgna@unina.it (M.L.); silvia.savastano@unina.it (S.S.); colao@unina.it (A.C.); cdisomma@unina.it (C.D.S.); 2 UNESCO Chair “Education for Health and Sustainable Development”, University of Naples Federico II, 80138 Naples, Italy

**Keywords:** cholesterol, lipids, Alzheimer’s disease, Parkinson’s disease, neurodegenerative diseases, statins, neurodegeneration, motor neuron disease

## Abstract

**Background/aim**: A growing body of evidence suggests a link between dyslipidemias and neurodegenerative diseases, highlighting the crucial role of lipid metabolism in the health of the central nervous system. The aim of our work was to provide an update on this topic, with a focus on clinical practice from an endocrinological point of view. Endocrinologists, being experts in the management of dyslipidemias, can play a key role in the prevention and treatment of neurodegenerative conditions, through precocious and effective lipid profile optimization. **Methods**: The literature was scanned to identify clinical trials and correlation studies on the association between dyslipidemia, statin therapy, and the following neurodegenerative diseases: Alzheimer’s disease (AD), Parkisons’s disease (PD), Multiple sclerosis (MS), and Amyotrophic lateral sclerosis (ALS). **Results**: Impaired lipid homeostasis, such as that frequently observed in patients affected by obesity and diabetes, is related to neurodegenerative diseases, such as AD, PD, and other cognitive deficits related to aging. AD and related dementias are now a real priority health problem. In the United States, there are approximately 7 million subjects aged 65 and older living with AD and related dementias, and this number is projected to grow to 12 million in the coming decades. Lipid-lowering therapy with statins is an effective strategy in reducing serum low-density lipoprotein cholesterol to normal range concentrations and, therefore, cardiovascular disease risk; moreover, statins have been reported to have a positive effect on neurodegenerative diseases. **Conclusions**: Several pieces of research have found inconsistent information following our review. There was no association between statin use and ALS incidence. More positive evidence has emerged regarding statin use and AD/PD. However, further large-scale prospective randomized control trials are required to properly understand this issue.

## 1. Introduction

Brain cholesterol is a basic constituent of neuronal membranes and it is the main component of synaptic vesicles [1,2]. On the postsynaptic side, cholesterol plays a remarkable role in the organization and the correct positioning of neurotransmitter receptors [2,3]. Lipids are also signaling mediators, thus acting as bioactive molecules called “bioactive lipids”, recognizing the main functions in the regulation of the immune system, inflammation, and maintenance of homeostasis [4]. Cholesterol is not uniformly distributed in cell membranes, but localized in lipid rafts [5]. Lipid rafts represent membrane microdomains enriched also in sphingolipid, able to modulate cell signaling and membrane fluidity, in order to regulate cellular processes and membrane trafficking of proteins. Neurotransmission is one of these main cellular processes [6]. Since cholesterol is a key component of lipid rafts also in the central nervous system (CNS), its depletion and disruption, as well as systemic cholesterol excess, could play a key role for disease-related cognitive impairment [7]. In recent years, an increasing number of genes involved in lipid metabolism have been implicated in several neurodegenerative diseases (NDDs) through Genome-wide association studies (GWAS). In particular, a dysregulation of lipid homeostasis can affect the aggregation and propagation of many pathogenic proteins that are well-known hallmarks of several NDDs, of which Alzheimer’s disease (AD) and Parkinson’s disease (PD) are representative pathologies [8]. In fact, the evidence of misfolded protein and genetic structures, so-called “cargo” [9], associated with extracellular vesicles (EVs) has led to the identification of their roles in intercellular communication and pathogenesis of NDDs such as PD, AD, and amyotrophic lateral sclerosis (ALS) [9]. These conditions share a pathogenic common mechanism, in which distinct proteins become misfolded and deposited in specific brain regions, suggesting the pathogenic disease process involves the intercellular movement of these proteins [10]. In recent decades, the relationship between cholesterol homeostasis and AD has been intensively studied. Zambòn et al. reported a higher incidence of cognitive impairment in familial hypercholesterolemia [11] and a high prevalence of hypercholesterolemia is found in subjects with cognitive decline, and no ethnic differences were found among Whites, African Americans, and Hispanics [12]. Accordingly, another pathogenic role of excess cholesterol in AD is to enhance the production of amyloid-β (Aβ), as the cholesterol in lipid rafts promotes the activity of g-secretase, the enzyme responsible for β-amyloid production [13,14]. Moreover, a Mendelian randomization study using 380 genetic variants associated with low density lipoprotein-cholesterol (LDL-C) levels as instrumental variables also suggested that low plasma LDL-C levels have a causal effect in reducing the risk of AD [15]. Excess cholesterol-induced toxicity is ubiquitous and “cholesterol toxicity” is a new concept that may help us better understand disease pathogenesis, including not only NDDs but also diabetes and liver diseases [16]. Patients affected by NDDs are characterized by the progressive loss of axons and neurons in the peripheral nervous system (PNS) and CNS. The effects are abnormality of cognition, impaired sensory and motor performance leading to remarkable disability, morbidity, and, frequently, premature mortality [17]. Certainly, NDDs result from complex neuropathological origins and are, consequently, difficult to treat. At present, there is a variety of efficacious therapies available, with a first “trigger” on symptomatic approach following disease onset. However, new therapeutic goals are focusing on strategies that target the early stages of neurodegeneration [18,19]. With these purposes in mind, an alternative therapeutic approach is to evaluate the potential of lipid-lowering molecules (LLMs), which act via different mechanisms to lower serum lipid concentrations [20]. Here, we review the effects of excess cholesterol on NDDs pathogenesis and discuss the possible effects of LLMs therapy in patients affected by such diseases. Although there is a growing body of evidence on this topic, the approach to this is often fragmented among different specialties. We decided to write this review to provide an endocrinological perspective on this topic, with a focus on clinical practice. Our aim is to provide an update based on the most recent scientific evidence, helping clinicians to identify patients at risk and to integrate new management strategies into their daily approach. Furthermore, we want to bridge the gap between research and clinical practice, emphasizing the role of the endocrinologist in a multidisciplinary context. Endocrinologists, being experts in the management of dyslipidemias, can play a key role in the prevention and treatment of neurodegenerative conditions, such as AD and PD, through lipid profile optimization and targeted therapeutic strategies.

## 2. Alzheimer’s Disease

AD is a progressive and irreversible neurological disorder that affects cognitive and functional abilities. It is considered the most common neurodegenerative disease and the most common cause of dementia in older adults [21,22]. Approximately 50 million people worldwide are currently living with the disease, and the number is expected to increase as life expectancy increases [23]. The risk of developing AD increases with age, and late-onset AD, which occurs in patients over age 65, is the most common form of AD. Early-onset AD is generally associated with familial forms of Mendelian genetics (familial AD, FAD). Other risk factors for AD include lifestyle factors (e.g., diet and lack of exercise) and certain diseases (e.g., hypertension and diabetes) [24]. The most characteristic symptoms of the disease are memory loss, speech difficulties, disorientation, and behavioral changes [25]. Although the exact etiology of AD remains unclear, these symptoms reflect the progressive death of brain cells and the resulting deterioration of cognitive function caused by the formation of plaques and tangles in the brain. Senile plaques result from the extracellular aggregation of Aβ due to its excessive production or impaired clearance, while tangles consist of twisted fibers of tau protein [26]. However, the exact mechanisms underlying the development and progression of the disease are not fully understood, so, alongside the many clinical trials targeting Aβ, research has focused on exploring other potential molecular mechanisms for AD. One line of research suggests that the metabolism of cholesterol and triglycerides may play a role in the development and progression of AD.

### 2.1. Lipids and Alzheimer’s Disease

After its recognition, the role of lipid accumulation in the affected brain has been studied with growing interest [27]. Animal studies have examined the link between lipids and AD. A high-fat diet may increase the risk of developing AD in animals. In fact, transgenic mice fed a high-fat diet have an increased risk of developing amyloid plaques, a hallmark of AD [28,29]. There is evidence of a relationship between high cholesterol levels in midlife and the development of dementia and in particular of AD [30] due to the observation that dyslipidemia, mainly high levels of LDL-C, has vascular and neurotoxic effects [31]. However, the link between hypercholesterolemia and AD risk remains unclear and is likely complex. For example, studies that examined the effects of midlife serum cholesterol on the risk of dementia and AD in later life have provided conflicting results [32,33]. The first one by Solomon et al. showed a positive association between serum cholesterol levels in midlife and the development of AD 21 years later [32], while in the study by Mielke et al., there were no significant association between serum cholesterol levels in midlife and the risk of AD 32 years later [33]. A common result between the two studies is still evident: a drop in serum cholesterol levels between midlife and late life is associated with a higher risk of developing AD. Another study by Mielke et al. found that hypercholesterolemia in late life reduces dementia risk [34] while, again in contradiction, no effect was found in the study by Li G et al. [35]. Finally, mild hypercholesterolemia has been associated with increased early amyloid plaque deposition in the brain, independent of the APOE genotype [36], but in the population-based Adult Changes in Thought (ACT) study, no association between late-life cholesterol and AD neuropathological changes was found [37].

A line of research focused on another group of lipids, which is sphingolipids (SPs), suggests that they could be implicated in the pathogenesis of AD, as they play a role in the formation and stability of cell membranes in the brain that are important for neuronal function [38]. Alterations in sphingolipid metabolism have been observed in the brains of people with AD, and studies suggest this may contribute to neuronal dysfunction and neurodegeneration [39]. Lipid metabolism may also be modified in the brains of animals with AD. For example, a 2004 study by Alessenko et al. found that the expression of genes involved in sphingolipid metabolism was altered in the brains of mice with AD [40]. In addition, mutations in genes involved in sphingolipid metabolism are associated with a higher risk of developing AD [41] and of having more severe forms of the disease [42].

Triglycerides (TGs) are a type of lipid found in the blood that play a key role in energy metabolism. Modifications in their metabolism may be associated with AD. First of all, data from preclinical studies in mouse models of AD with abundant plasma Aβ show that elevated plasma TGs levels precede amyloid deposition, suggesting that high TG levels may contribute to the development and progression of AD [43]. Dietary interventions that target TG metabolism may have the potential to treat AD. For example, a 2022 study by Amelianchik et al. [44] found that mice with AD that were fed a diet enriched with a certain type of fatty acid had better cognitive function and formed fewer amyloid plaques [44]. Several clinical studies have examined the association between TGs and AD, with results concluding that high TG levels in midlife may increase the risk of developing the disease later in life and, consequently, that serum TG levels can be useful biomarkers for AD. A cross-sectional study on normal cognitive individuals showed that a high serum TG level was associated with an increased global amyloid-PET signal after APOE4 adjustment, while no correlation was found for total cholesterol, high density lipoprotein–cholesterol (HDL-C), or LDL-C. However, Aβ deposition in medial temporal, occipital, and basal ganglia regions was not positively associated with high serum TG [45]. Another longitudinal cohort study of 318 cognitively normal individuals concluded that increased levels of TG at midlife predict brain Aβ and tau pathology 20 years later in cognitively healthy individuals, after adjusting for multiple vascular factors, education, age, and APOE4. Some lipoprotein subfractions may also be risk factors for Aβ pathology [31]. The longitudinal study with over 10 years of follow-up (the Framingham Heart Study) was conducted on 157 cases and 2882 controls with AD status and genotypes available and aimed to explore the interaction of a genetic risk score (GRS) of AD risk alleles with mid-life plasma lipid levels (LDL-C, HDL-C, and TG) on the risk for AD. The results showed that there was an influence of hypertriglyceridemia during midlife (40–60 years of age) on late-onset AD risk in APOE e4 negative participants, after adjustment for systolic blood pressure and based on genetic markers [46]. However, less encouraging evidence comes from other studies. Reed et al. [47] conducted a small cross-sectional study on 74 individuals (3 with mild dementia, 38 with mild cognitive impairment, and 33 clinically normal) to investigate the association between serum cholesterol levels and cerebral Aβ measured with carbon C11-labeled Pittsburgh Compound B (PIB) positron emission tomography during life early in the AD process. No correlation was found, although there was an independent association between LDL-C, HDL-C, and amyloid brain deposition [47]. Proitsi et al. found an association of low-chain and very-low-chain TG with AD in the untargeted lipidomic analysis on 142 AD and 152 control subjects [48]. Finally, evidence from a small sample cross-sectional study has shown a decreased TG level in dementia subjects. The study outlined the presence of lower TG levels in probable AD individuals (24 females and 6 males) compared to control groups [49]. Overall, the results of animal and clinical studies are inconclusive about the role of lipids, particularly cholesterol, sphingolipids, and TG, in the development and progression of AD. Further research in this area may ultimately lead to the development of new strategies to prevent or treat this devastating disease. Some encouraging data on the use of statins are already known, although the evidence does not always agree.

### 2.2. Statin Use and Alzheimer’s Disease

Several studies have examined the association between statin use and AD, and there is some evidence that statins may be used to treat AD (Table 1). The protective effects of statins in preclinical studies have been very consistent. The drugs have been reported to reduce Aβ levels in vitro, through the activation of the metallopeptidase ADAM10 and the increase in phospholipid transporter (PLTP) activity, leading to a decrease in p-tau181 [50,51]. Results are also promising in mouse models, since therapy with atorvastatin and pravastatin proved to be capable of reducing the Aβ-plaque burden and microglial inflammation in transgenic AD mice [52], while simvastatin can improve cognitive deficits in mice without altering the amyloid plaque burden [53,54]. Simvastatin, atorvastatin, and ezetimibe have been shown to be effective in reducing the neurofibrillary tangle (NFTs) burden in a mouse model of tauopathy [55]. In contrast, the results of clinical trials are more controversial. While early cross-sectional studies have shown that statin use can reduce the risk of AD by up to 70% [56,57], prospective longitudinal studies later provided mixed results. A cohort study of 2798 individuals found no association between statin therapy and a lower risk of dementia [58], while in the study by Cramer C et al. on 1789 older Mexican Americans, statin users were less likely to have an incident of dementia/cognitive impairment without dementia during a 5-year follow-up [59]. Results from randomized controlled trials (RCTs) [60,61] suggest that statins may be useful in slowing the progression of AD when it is already manifest, but not in those who are at risk or who have a mild expression of the disease. In fact, two large RCTs, the Heart Protection Study [60] and the PROspective Study of Pravastatin in the Elderly at Risk (PROSPER) [61], which examined the effects of statins in the non-demented elderly at high cardiovascular risk, concluded that statins do not have a significant protective effect on cognition. More promising results in cognitive, functional, and behavioral outcomes came from the Alzheimer’s Disease Cholesterol-Lowering Treatment (ADCLT) trial, which compared atorvastatin (80 mg/day, *n* = 32) with a placebo (*n* = 31) in a small sample (*n* = 63) of patients with mild to moderate AD dementia [62], but these results were not confirmed in longer and larger clinical trials, such as the Lipitor’s Effect in Alzheimer’s Dementia (LEADe) trial [63]. In this international, multicenter, double-blind, randomized, parallel-group study, 640 patients with mild to moderate AD dementia were randomly assigned to atorvastatin (80 mg/day, *n* = 297) or a placebo (*n* = 317). After 72 weeks, no benefit in cognition and global functioning were observed between the groups. In a randomized, double-blind, placebo-controlled trial conducted in 406 individuals with mild to moderate AD and normal lipid levels, simvastatin (40 mg/day) also had no effect on the rate of cognitive decline [64]. The effect of statins on AD may depend on the type of statin used: lipophilic statins have been shown to attenuate the progression of AD from mild to moderate, possibly because lipophilic statins are more likely to cross the blood–brain barrier (BBB) [65], whereas the hydrophilic pravastatin has no such effects. The lipophilic simvastatin and lovastatin have been widely used to improve memory and learning ability. Overall, the evidence on the relationship between lipids and AD and the potential role of statins in the prevention and treatment of this form of dementia is promising, but further research is needed to draw more meaningful and definitive conclusions.

## 3. Parkinson’s Disease

PD is a neurological illness marked by the progressive loss of monoaminergic cell types in the brainstem, particularly dopaminergic neurons in the substantia nigra pars compacta. PD is characterized by diminished levels of dopamine in the nigrostriatal pathway, which leads to postural and gait problems, bradykinesia, stiffness, and resting tremor [66,67]. While the precise etiology of PD remains unclear, it seems to arise from a complicated interplay between hereditary and environmental variables that impact several essential cellular processes. Proteasome failure, immunoinflammatory reactions, oxidative stress, and mitochondrial dysfunction are examples of potential processes [66,67]. The lipid alterations in patients with PD were found to be associated with insulin resistance, oxidative phosphorylation/thermogenesis, and the metabolism of glycerolipids, phospholipids, and sphingolipids [68]. Parkinsonism has been associated with either autosomal dominantly (α-synuclein, LRRK2, VPS35, EIF4G1) and recessively (PARK2, PINK1, DJ-1, SYNJ1, and PLA2G6) inherited mutations [69]. Furthermore, the largest known genetic risk factor for PD development is heterozygous mutations in the glucocerebrosidase (GBA) gene [70]. Lipids play a role in many distinct aspects of PD pathology. These include modifications to metabolism or lipid pathways in PD patients or research models, specific cytotoxic interactions with PD-causing genes, and enzyme mutations that significantly enhance the risk of PD [71].

### 3.1. Lipids and Parkinson’s Disease

Sphingolipids play a key role in the development of PD [72,73,74]. Recent studies have shown a selective decrease in ceramide (CER) and sphingomyelin (SM) and bioactive sphingolipids in brain regions affected by PD pathology [75]. Moreover, genetic studies have indicated the potential involvement of sphingolipids in PD pathogenesis [76,77,78]. Plasma ceramide and sphingomyelin levels in idiopathic PD patients were associated with clinical symptoms and dopaminergic degeneration, the hallmark of PD. Lower plasma ceramide and sphingomyelin levels are potentially associated with faster progression in clinical presentations. CER and SM regulate the postsynaptic function in the striatum, influencing dopamine release and absorption via the ceramide/sphingomyelin pathway [79]. Sphingolipid changes appear to affect lipid membrane architecture, such as integral protein placement, which may influence neuronal activities, such as dopamine receptor binding [80]. Anatomical and physiological evidence suggests that sphingolipids regulate dopamine transporter reuptake via the sphingomyelin pathway [81].

While the role of sphingolipids in the pathogenesis of PD is well known, the literature data for the other components of the lipid profile are not well-defined. First of all, results on the relationship amongst plasma HDL-C and PD were found to be contradictory. Higher levels of TG, total, and LDL cholesterol may be protective in the genesis of PD, despite the lack of evidence of a link with HDL-C [82]. Increases in total and LDL-C, TG, and apoB, but not HDL-C, were consistently associated with a lower risk of PD development in the AMORIS study [83]. This could be the result of a decline in the neuroprotective effects of coenzyme Q10 of lowering serum cholesterol levels [84]. Coenzyme Q10’s strong neuroprotective qualities and capacity to lower oxidative stress have been connected in animal research to the onset of PD. It might also postpone the depletion of dopamine [13]. Several rat studies have supported the preventive function of cholesterol precursors in the pathophysiology of PD. The detrimental effects of 6-hydroxydopamine on striatal neurons may be inhibited by squalene [85], whereas lanosterol acted protectively against 1-methyl-4-phenyl-1,2,3,6-tetrahydropyridine toxicity on dopaminergic neurons in the substantia nigra in mouse models of PD [86]. Numerous studies have shown a possible connection between reduced serum TG levels and PD risk [82]. Sympathetic activity in PD patients was similarly reduced [87], and TG levels significantly dropped as a result of reduced cortisol and catecholamine synthesis. Because non-motor symptoms have historically preceded motor symptoms, lower cholesterol levels may result from significant sympathetic denervation or autonomic problems before PD is clinically diagnosed [88,89]. Furthermore, PD patients who take levodopa as a prescription are more likely than other PD patients to experience peripheral effects from the drug’s conversion of dopamine, which may lower TG levels [90].

### 3.2. Statin Use and Risk of Parkinson’s Disease

Statin use was found to considerably reduce the incidence of PD by 26%, and Zhiguo Sheng et al. also found no correlation between LDL-C levels and the protective effects of statins against PD [91]. These findings were consistent with past meta-analyses conducted by Undela K et al. [92] (Table 1). Numerous mechanisms, including oxidative stress, neuroinflammation, and mitochondrial failure, are thought to be involved in the pathogenesis of PD. The strong anti-inflammatory effects of statins have been demonstrated [93,94]. Interestingly, another study discovered that using statins could increase dopamine in the striatal area levels in animal models of PD [95]. Furthermore, according to additional research, statin treatment lowers plasma cholesterol, which in turn reduces α-synuclein aggregation in PD patients [96]. When combined, statins may reduce neuroinflammatory responses, thereby safeguarding.

## 4. Multiple Sclerosis

The CNS neurodegenerative condition known as multiple sclerosis (MS) is characterized by persistent inflammation and demyelination [97]. Women are diagnosed with MS two to three times more frequently than males, and most cases happen between the ages of 20 and 50 [98].

It is a multifaceted, intricate immune-mediated illness that is influenced by both inherited and environmental variables. Relapsing-remitting MS and clinically isolated conditions are examples of the transitory bouts of neurological impairment that often last for many days or weeks during the early stages of the disease. Over time, clinical and cognitive issues become irreversible. Only a small fraction of people has an illness that worsens with time. The pathological hallmark of MS is the appearance of demyelinating plaques in the brain and spinal cord that can be accompanied by neuro-axonal degeneration [99]. Focal lesions are thought to be the result of immune cells (T and B cells) and myeloid cells infiltrating the parenchyma of the central nervous system and causing damage [98].

### 4.1. Lipids and Multiple Sclerosis

The theory that MS could be connected to a change in lipid metabolism has been prevalent for a while. The changes in the plasma lipid composition are caused by an unclear pathogenetic processes. However, it is well-established that the development of MS plaques is linked to the activation of a number of inflammatory mediators, including cytokines and anaphylatoxins, which alter the liver’s capacity to manufacture plasma lipoproteins [100]. Many of these lipoproteins have higher plasma levels when MS is active, and some of them, like serum Aβ, have an impact on the metabolism of cholesterol [101,102]. There was shown to be a substantial correlation between the prevalence of this disorder and LDL-C and total cholesterol levels [103]. There is evidence that LDL-C plays an early role in the development of MS lesions: oxidative modification within the lesion and the breakdown of the blood–brain barrier allow a significant proportion of plasma LDL to reach the MS plaque parenchyma [104]. In the early phases of MS plaque, oxidized LDL absorption by invading macrophages and lipid peroxidation may have a substantial impact on demyelination [104]. The total cholesterol in plasma may be considered a biological marker of disease activity in patients experiencing their first demyelinating episode. Ascertained by MRI, it is sensitive enough to detect disease activity and easy to evaluate using conventional lab techniques. However, more investigation is needed to clarify the pathogenetic mechanisms influencing the plasma lipid profile during the acute phase of the disease and determine how much plasma levels of total cholesterol and LDL-C are helpful in practical MS disease tracking over an extended period of time [104]. MS is linked to dyslipidemia and a modified blood lipid profile (high LDL-C/low HDL-C). Throughout childhood and adolescence, a high body mass index (BMI) has been associated with an increased risk of multiple sclerosis. It has also been previously reported that there are some associations between BMI and markers of clinical progression in MS patients, despite notable differences among studies [105]. Individuals with raised TC, LDL, non-HDL, and triglycerides, as well as a bad lipid profile, were found to have higher levels of clinical impairment [106].

### 4.2. Statin Use and Risk of Multiple Sclerosis

Dyslipidemia is observed as one of the imperative risk factors involved in MS neuropathology, and chronic inflammation in MS predisposes to the progress of dyslipidemia. Lipid-lowering medications have also been investigated in this illness (Table 1). A 2017 study [107] found that a high dose of simvastatin improved the physical quality of life and frontal lobe function in patients with secondary progressive MS. This is consistent with other research that has examined the impact of blood lipids on the cognitive impairment of MS patients [108,109]. In a 2023 review, Hayder M Al-Kuraishy et al. reported the correlation between the statin’s anti-inflammatory and immunomodulatory properties and their protective effect against the acute phase of MS. However, treatment with statin in the chronic phase of MS could have detrimental effects by reducing brain cholesterol and inhibiting the remyelination process [110].

Given that statins therapy could be effective with minimal complications for patients affected by MS, a molecular marker is necessary for treatment response evaluation. In 2023, an uncontrolled study was published by Batoee S et al. [111], which aimed to evaluate SIRT1 gene expression changes following rosuvastatin therapy in patients with MS. SIRT1 was significantly upregulated in MS patients after 3 months of rosuvastatin therapy. Subsequently, The Expanded Disability Status Scale (EDSS) of patients was decreased, along with the increase in SIRT1 gene expression, although EDSS changes were not significant, probably due to the treatment’s short duration. In fact, EDSS is a late response. The authors finally stated that SIRT1 could be a sensitive and reliable biomarker for early evaluation of the MS treatment response. Finally, several RCTs of statins, in any form or dosage, as monotherapy or add-on to established therapy in relapsing-remitting MS (RRMS), progressive MS, and clinically isolated syndrome are actually ongoing [112]. In particular, the MS-STAT2 trial will be the first phase 3 randomized controlled trial to assess the effectiveness of repurposed simvastatin compared with a placebo in slowing the progression of disability in secondary progressive MS as a potential neuroprotective agent [113].

## 5. Amyotrophic Lateral Sclerosis

Lou Gehrig’s disease, or amyotrophic lateral syndrome (ALS), is a degenerative neurological condition marked by the gradual degeneration of both the central (cortex) and peripheral (spinal cord and somatic motor nuclei of the cranial nerves) motor neurons [114]. The condition has a gradual onset, first showing up as a muscular weakness linked to atrophy, fasciculation, and weight loss. However, additional muscle functions quickly disappear, including dysarthria and gradual paralysis, ultimately leading to the failure of the respiratory system, which is the primary cause of death. The condition progresses quickly, and death usually happens one to five years after ALS onset [114]. Ten percent of cases are hereditary, usually linked to gene mutations (SOD1, TDP-43, FUS and C9ORF72) while the remaining ninety percent are sporadic (unknown origin) [115].

The cause is unknown; however, a number of theories have been proposed thus far, the most significant of which are the oxidative [116], excitotoxic [117], and neuroinflammatory [118] ones. There is now no treatment available; however, by elucidating the pathogenic mechanism, possible therapeutic targets may be found to ameliorate the dismal prognosis.

### 5.1. Lipids and Amyotrophic Lateral Sclerosis

ALS’s onset, course, and prognosis are all undisputedly correlated with blood lipid levels. There is evidence to support the concept of lipid-mediated toxicity meaning that the lipid imbalance in the CNS and blood of ALS patients is clinically associated with the extent of the disease, functional decline, and lifespan [119]. The etiology of ALS may also involve another aspect of lipid chemistry, namely lipid peroxidation, which increases fatty acid saturation. Patients with ALS have been shown to have significantly greater amounts of unsaturated TG, according to an examination conducted using mass spectrometry [120]. The worst muscle performance may result from neuronal injury brought on by the production of oxidized metabolites of excess cholesterol and lipid peroxidation [121]. Numerous studies demonstrate that patients with ALS who have high TG live longer because raised serum TG promotes neuron survival [122,123,124]. Wuolikainen et al. discovered a gender impact, finding that ALS patients who were female had greater TG levels than those who were male [125]. Moreover, TG levels may have an impact on an ALS patient’s longevity because they are a measure of nutritional status, and it is commonly recognized that hypercatabolism and losing weight occur in the latter stages of the disease [126]. The role of membrane cholesterol in ALS processes is a topic that is not well covered in the published literature. An increasing body of research indicates a beneficial correlation between ALS survival and elevated plasma cholesterol levels [127]. The survival rate was enhanced by more than a year in patients with elevated LDL/HDL ratios [128]. The overall survival from onset has been shown to be predicted by HDL-C in multiple investigations [129,130]. Given that HDL indicates nutritional status and that a lower BMI is associated with higher HDL, elevated HDL concentrations and BMI are inversely connected [122,131]. More recent research by Mark et al. showed that higher clinical stages are associated with lower serum concentrations of LDL-C and total cholesterol [131].

Current research suggests that a dysregulated sphingolipid metabolism may be a significant modulator of the disease course in ALS, being connected to the neurodegenerative process. Both the spinal cords of ALS patients and the spinal cords of asymptomatic and symptomatic SOD1 G93A mice have been shown to have an aberrant rise in sphingolipid concentrations [132]. The spinal cords of Sod1 G86R mice [133] and SOD1 G93A mice, as those of patients affected by ALS [134], have been shown to have higher levels of sphingolipid metabolites (ceramide, GM1, GM3, glucosylceramide, galactosylceramide).

### 5.2. Statin Use and Amyotrophic Lateral Sclerosis

Based on multiple pieces of research, there are inconsistent data about the effect of statins on the likelihood of ALS. According to a 2021 PRISMA-compliant meta-analysis [135] (Table 1), there is no link between the usage of statins and the incidence of ALS. A 2017 nation-wide, population-based case-control study indicated an association between statin use and a lower risk of ALS [136], while, on the contrary, a harmful effect emerged in a Mendelian randomization analysis by Wang et al. [137]. Concerns regarding the security of statins in ALS have been raised by reports of increased muscular cramps and functional impairment among ALS patients using them [138]. Recently, a 2025 Norwegian population-based cohort study [139], showed no association between statin use and ALS survival, confirming the results of two previous case-control studies [140,141]. Further large-scale, prospective, randomized control studies are necessary, nonetheless, in order to draw clear results.

**Table 1:** Description of the principle studies that correlate statin therapy and the clinical course of neurodegenerative diseases.

Authors’ Conclusions	Neurological Disease	Population of the Study	Type of Study	Aim of Study	Reference
The cohort taking statins during the study period had a 60% to 73% (*p* < 0.001) lower prevalence of probable AD.	Alzheimer’s Disease	57,104 total patient from 3 different hospitals	Cross-sectional analysis	The aim of the study was to compare the prevalence of AD in patients 60 years or older in the following 3 groups: (1) the entire population; (2) patients receiving statins; (3) patients receiving medications used to treat hypertension or cardiovascular disease.	Wolozin B. et al., 2000 [56]
Treatment with atorvastatin could have a potential clinical benefit in patients with AD.	Alzheimer’s Disease	63 participants with mild to moderate Alzheimer’s disease: -31 assigned to placebo -32 assigned to 80 mg atorvastatin time of observation: one year	Placebo-controlled randomized trial	The primary outcome measures were change in Alzheimer’s Disease Assessment Scale–cognitive subscale [ADAS-Cog] and the Clinical Global Impression of Change Scale scores [ADCS-CGIC].	Sparks DL et al., 2005 [62]
Atorvastatin was not associated with significant clinical benefit over 72 weeks. However, patients enrolled did not need statin treatment, due to their good lipidic profile.	Alzheimer’s Disease	640 participants with mild to moderate Alzheimer’s disease: -326 assigned to placebo -314 assigned to 80 mg atorvastatin/day Time of observation: 72 weeks followed by a 8-week atorvastatin withdrawal phase	Multicenter, double-blind, randomized	Coprimary endpoints were changes in cognition [ADAS-Cog] and global function [ADCS-CGIC]) at 72 weeks.	Feldman HH et al., 2010 [63]
Early statin use was significantly associated with a reduction in AD progression in mild-to-moderate AD patients.	Alzheimer’s Disease	23.074 million people (total population of Taiwanese citizens seen in general medical practice) Time of observation: one year after starting use of any acetylcholinesterase inhibitors	Case-control study	Alzheimer disease patients with early statin use (before AChEI treatment) were those receiving any statin treatment during the exposure period. The primary outcome was the discontinuation of AChEI treatment, indicating AD progression.	Lin F-C et al., 2015 [65]
Statin use reduces the risk of PD.	Parkinson’s Disease	1,457,836 participants without PD 15,102 participants with PD	Meta-analysis of: 5 case–control studies 3 cohort studies	The aim was to study the association between statin use and risk of developing PD.	Undela et al., 2013 [92]
High dose of Simvastatin had a positive effect on frontal lobe function. No other significant effects on the neurological outcome.	Multiple sclerosis	140 patients with secondary progressive multiple sclerosis (SPMS): -70 assigned to placebo -70 assigned to 80 mg simvastatin Time of observation: 24th months	Clinical trial	The aim of the study was to investigate the effect of high-dose simvastatin on cognitive, neuropsychiatric, and health-related quality-of-life (HRQoL) outcome measure in patients with SPMS. Assessments were conducted at study entry, 12 months, and 24 months.	Chan et al., 2017 [107]
No definite association was found between statin use and the development of ALS.	Amyotrophic Lateral Sclerosis	11,747 participants with ALS 230,573 participants without ALS	PRISMA meta-analisis: 3 case-control studies and 1 cohort study	The incidence of ALS in statin- and non-statin-treated patients was measured. The principal aim was to determine the effect of statins on ALS incidence.	Chang MC et al., 2021 [135]

## 6. Genetic Mutations and Neurodegenerative Diseases: Correlation with Lipid Metabolism

Genetic studies have identified mutations in lipid metabolism-associated genes such as glucocerebrosidase 1 (GBA1) in sporadic PD and synaptojanin 1 (SYNJ1) in familial forms of PD [142]. PD is characterized by Lewy bodies (LBs) composed mainly of an aggregation of α-synuclein (αSyn), which is also a characteristic feature of dementia with Lewy bodies (DLB) [143]. In physiological conditions, αSyn is involved in fatty acid metabolism in the brain and it was reported to interact with the lipid membrane [142,144,145]. The interaction between αSyn and lipid membrane appears to induce the aggregation of αSyn, implying the possible roles of lipids in the pathogenesis of PD [142,146,147]. Several studies, have demonstrated that lipid’s dysregulation by GBA1 and SYNJ1 mutations induces the aggregation of wild-type αSyn (wt) [142,148,149,150]. Furthermore, the expression of either wt-or mutant αSyn induces neurodegenerative alterations via the accumulation of αSyn aggregates, with consequent motor function deficits [142,150,151]. Therefore, the interaction between lipids and αSyn represent one of the key factors in inducing αSyn aggregation. These results suggest the central role of αSyn in the pathogenesis of familial and sporadic PD [142,146]. Probably, the control of interaction between αSyn and lipids by small molecules would be an innovative opportunity in the therapeutic algorithm for patients affected by PD [142].

ALS is a neurodegenerative disease characterized by the progressive degeneration of motor neurons in the primary motor cortex, brainstem, and spinal cord, leading, at first, to skeletal muscle atrophy and, subsequently, paralysis. Two genes, C9orf72 and SOD1, are mainly involved in familial ALS (10% of cases). For sporadic ALS (90% of cases), the cause of the disease is unknown [152]. Several cellular pathways, such as oxidative stress, mitochondrial dysfunction, and RNA metabolism alterations, are linked to neurodegeneration in ALS [152,153,154]. Currently, in skeletal muscle, the role played by alterations in lipid and carbohydrate metabolism in the pathogenesis of ALS is well-known [152,155,156,157]. At present, dyslipidemia represents one of the main metabolic alterations that characterizes ALS; however, its role in the pathophysiology of the disease remains unclear. Recently, a higher LDL/HDL cholesterol ratio was correlated with longer survival [130,157], while previous data found no correlation between serum lipid alterations and ALS survival [128,158]. At first, some studies reported a protective role of hypercholesterolemia detected prior to the onset of motor symptoms [159], while the protective role of hypercholesterolemia was not subsequently confirmed [160]. On the contrary, in another study, a higher LDL/HDL cholesterol ratio and increased serum levels of LDL cholesterol were associated with a higher incidence of ALS [160]. Finally, genome-wide association studies (GWAS) suggest a positive correlation between LDL cholesterol and the risk of ALS [161,162,163].

Very recently, Delphine Sapaly et al. [152] found that in the skeletal muscle of patients affected by ALS, cholesterol accumulates significantly, correlating with severity of the disease. In particular, there was the detection of an overexpression of the two genes of the lysosomal cholesterol transporters, Niemann-Pick type C1 (NPC1) and 2 (NPC2), which are both involved in cholesterol transfer mechanisms. Moreover, a significant increase in NPC2 mRNA levels was found in muscle samples even long before the onset of the disease [152]. Finally, following acute NPC1 inhibition, in human control myotubes, a preferential use of fatty acid was reproduced, similarly to metabolic alterations that characterize ALS [152]. Therefore, the authors have concluded that cholesterol homeostasis is dysregulated in ALS muscle from the presymptomatic stage and that targeting NPC1/2 alterations may represent a new therapeutic approach for patients affected by ALS [152]. Although ALS may be considered as a single pathology, it really should be considered as a complex group of diseases, characterized by multiple interactions between several genetic mutations and different metabolic pathways [164].

AD is considered the most frequent type of neurodegenerative disease, occurring in 60% to 80% of all cases [165]. In familial AD, mutations in the APP, PSEN1, and PSEN2 genes induce an early onset of AD (EOAD). On the contrary, sporadic AD affects adults over 65 years of age and is, therefore, classified as late-onset AD (LOAD) [166]. In patients affected by LOAD, the gene involved is APOE [166,167], but many subjects with the APOE ε4 allele do not develop AD, indicating, probably, the involvement of other genetic risk factors. Certainly, AD is caused by more than one gene, and now it is complicated to predict the age of onset during presymptomatic diagnostic tests [168]. In recent years, several genes, also called susceptibility genes and associated with LOAD, have been identified; probably, they can increase the susceptibility of each subject to developing AD. However, these are novel genes and, actually, their global function remains unknown [166]. The identification of gene mutations can help for an early diagnosis and better management of AD in terms of clinical features, imaging, and pathology of this disease. On these bases, genetic data can help also to provide necessary information in order to start clinical trials for therapeutic strategies [169] and to design new clinical trials for preventing AD and /or delaying the disease [166,170]. Indeed, ongoing clinical trials will determine whether the therapeutic options administered in the preclinical and/or asymptomatic phase help in delaying or preventing the progression of the disease. It is well-known that correct nutrition and adequate motor activity play a significant role in modulating the effects of aging, which probably delays and/or reduces the incidence of AD [166]. Furthermore, pathologies such as diabetes, being overweight or obesity, hypertension, and dyslipidemia itself should be recognized and treated early, in order to reduce not only cardiovascular disease but also the risk of AD [166].

The genomic map provides clear evidence that MS is a principally neuroinflammatory disease that induces demyelination and neuronal injury and then neurodegenerative disease [97]. Previous data had already indicated the genetic linkage between MS and the chromosome 19q13 region [171]. ApoE, a cholesterol transport protein, influences several pathways related to neurodegeneration also in MS [172]. In particular, in patients affected by MS, the influence of ApoE4 on the pathogenesis of the disease appears predominantly adverse [173]. The ApoEε4 allele in patients affected by MS suggest that it may influence disease progression and consequently, the severity of the disease. In particular, ApoE4 and the other two primary human ApoE polymorphisms, ApoE2 and ApoE3, modulate the redistribution of phospholipids and cholesterol to neurons through their interaction with cell-surface ApoE receptors. On these bases, ongoing ApoE studies underscore the possibility for pioneering therapeutic strategies in order to reduce the progression of neurodegenerative diseases also in MS [173].

## 7. Autophagy, Lysosomal Degradation, and Effects on Lipid Metabolism

Autophagy is a multistep and sophisticated cellular process, responsible for the degradation and recycling of cytoplasmic contents. This mechanism involves numerous proteins and lipids derived from diverse membrane sources, such as endoplasmic reticulum (ER), Golgi, plasma membrane, recycling endosomes, and endoplasmic reticulum/mitochondria contact sites [174]. Therefore, autophagy represents a conservative mechanism that degrades damaged and/or superfluous cellular contents [175]. Lipids are constantly recycled and thus redistributed within a cell. In this recycling mechanism, lysosomes play a significant role via autophagy or endocytosis [176]. This is a bidirectional mechanism; in fact, the lysosomal degradation process regulates cellular lipid metabolism, whereas lipids also regulate lysosome function and autophagy [177]. Neurons are strongly vulnerable to autophagy dysregulation because they cannot dilute damaged substances and/or structures through mitosis [175]. Mitochondria represent the main source of cellular energy used to regulate metabolic processes [178] and they modulate the synthesis and the deployment of neurotransmitters mechanisms, dependent, principally, on calcium regulation and ATP generation via Oxidative Phosphorylation [179]. Alterations in lipid metabolism induce mitochondrial dysfunction and a stress response in the ER; in particular, changes in lipid content, increasing oxidative stress, can accelerate mitochondrial dysfunction in neurodegenerative diseases (NDDs) [180,181]. In particular, Xin Li et al. already have shown that ELOVL fatty acid elongase 2 (Elovl2), a gene whose epigenetic alterations are highly correlated with age prediction, contributes to aging by regulating lipid metabolism [180].

Several data showed that autophagy plays a protective role against the development of insulin resistance and diabetes [182]. Significant is the removal of dysfunctional mitochondria via mitophagy, an aspect of autophagy selective for mitochondria. Muscle insulin resistance and the dysfunction of the β-cell strongly depend on the metabolic overload of mitochondria, which induces an oxidative stress and the accumulation of toxic lipid intermediates with mitochondrial damage. Mitophagy eliminates oxidative stress and the resulting mitochondrial damage, counteracting pathogenic processes. In adipose tissue, autophagy induces adipocyte differentiation, likely for mitochondrial clearance. In addition, in diabetic conditions; autophagy protects β cells against ER stress and modulates exercise-induced increases in muscle glucose uptake. On these bases, autophagy/mitophagy mechanisms represent an interesting therapeutic target for obesity and diabetes [182]. Yang JS et al. have demonstrated the correlations between and development of various diseases, including diabetes mellitus, neurodegenerative changes, and aging [183]. In particular, in patients affected by type 2 diabetes (T2D), impaired autophagy in pancreatic β-cells plays a significant role in β cells’ dysfunction. Autophagy represents a key control system for maintaining β cells’ quality and glycemic level control [184]. On these bases, autophagy represents a significant potential as a new therapeutic approach for diabetes treatment [184] in order to improve therapeutic outcomes and quality of life for diabetes patients.

## 8. Obesity and Neurodegenerative Diseases

Obese patients frequently have abnormalities in their lipid metabolism. Dyslipidemia affects between 60 and 70 percent of obese persons [185]. Elevated blood TG, VLDL, apolipoprotein B, and non-HDL-C levels are among the lipid abnormalities seen in obese people. Increased hepatic synthesis of VLDL particles and decreased clearance of TG-rich lipoproteins are the causes of the rise in serum TG. Usually low, HDL-C levels are linked to elevated blood TG levels. Although there is a rise in small, dense LDL, LDL-C values are often within the normal range or only slightly higher [186]. Obesity has been linked to an increased risk of dementia, regardless of T2D [187]. Chronic low-grade inflammation associated with obesity in middle age offers a mechanistic link to progressive cognitive deterioration via cross-talk with cerebral inflammation [188]. Obesity is often linked with low-grade inflammation due to enhanced infiltration and the activation of innate and adaptive immune cells in peripheral tissues, such as adipose tissue [189,190], which can contribute to neuroinflammation [191,192]. Circulating inflammatory substances can spread peripheral inflammation, activate macrophages in other organs, including the liver and pancreas, and can cause brain inflammation [192,193,194]. Obesity can increase BBB permeability, making the brain more susceptible to inflammation [195]. In neuroinflammation, proinflammatory and neurotoxic mediators are released into the brain, and microglia and astrocytes are activated [196,197]. Originally a defense mechanism, excessive neuroinflammation can cause neurodegenerative illnesses such as AD, MS, and PD [198,199], as well as neuronal dysfunctions [189,192]. A number of experimental animal models have proposed a causal link between neurodegenerative disorders and obesity. In a PD animal model, overnutrition can directly encourage dopaminergic neurodegeneration [200]. A key inflammatory modulator is the IKKb/NF-kB pathway, which regulates apoptosis and cell survival. For instance, it has been demonstrated that interleukin-6, a cytokine that is overproduced in patients with diabetes and obesity, mediates the degeneration of GABAergic interneurons in the forebrain. This neurodegenerative effect has been linked to the activation of neuronal NF-kB and the resulting induction of neurotoxic pro-inflammatory products [201]. Moreover, metabolic excess and neuronal resistance to insulin are linked to both obesity and type 2 diabetes, yet both conditions make neurons susceptible to cell death through inflammation and brain stress [202,203]. All things considered, neurodegeneration brought on by neuroinflammation may serve as a common foundation for both neurodegenerative and metabolic disorders, including obesity and type 2 diabetes. Furthermore, intestinal permeability may be increased by obesity-induced gut dysbiosis, resulting in a “leaky gut” and the increased release of lipopolysaccharides (LPS) that could exacerbate inflammation [204].

## 9. Discussion and Conclusions

**Figure 1:**
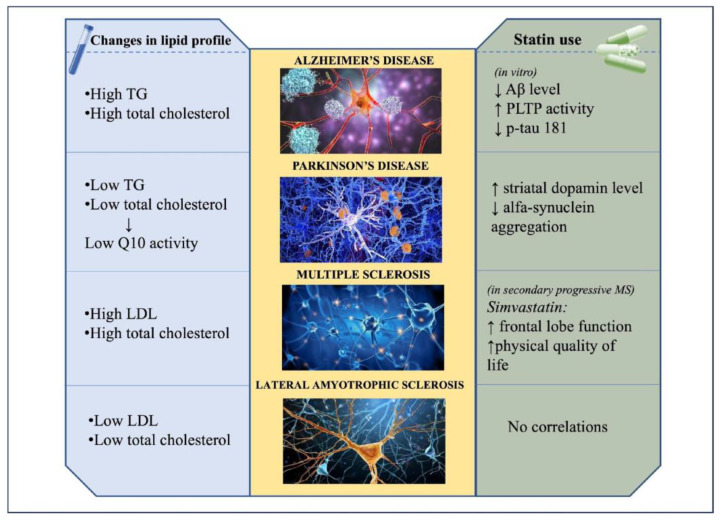
Summary of changes in lipid profile and effects of statin use in Alzheimer’s disease (AD), Parkinson’s disease (PD), Multiple sclerosis (MS), and Lateral amyotrophic sclerosis (ALS). The pathological hallmarks of the diseases are also depicted: AD: beta-amyloid and tau aggregates; PD: synuclein inclusions; MS: demyelination; ALS: FUS/SOD1/Tdp-43 aggregations.

A summary of the evidence that emerged from our review on the changes in the lipid profile and effects of statin use in AD, PD, MS, ALS is shown in Figure 1.

Indeed, the mechanistic links between specific lipid metabolism parameters and neurodegenerative disease remain controversial. In a very recent study [205], in order to assess potential causal links between neurodegenerative disease and lipid parameters, two-sample Mendelian randomization (MR) was used. This approach allowed us to overcome several limitations of observational studies and provided robust evidence for causal associations [206,207]. Understanding these correlations could have significant implications for disease prevention and/or treatment approaches. In the study of Zeyu Wang et al., the results (MR via the inverse-variance weighted method) showed the causal effects of cholesterol (CHOL, OR = 1.10, 95% CI: 1.03–1.18, *p* = 4.23 × 10^−3^) and low-density lipoprotein cholesterol (LDLC, OR = 1.10, 95% CI: 1.03–1.17, *p* = 3.28 × 10^−3^) on the risk of ALS, and these results were validated across multiple methods. Potential correlations were observed between ApoB and ALS; moreover, inverse correlations were demonstrated with AD, and no significant associations were found in patients affected by PD and MS. Cholesterol and LDLC associations with ALS demonstrated no significant heterogeneity or pleiotropy, supporting their reliability [205].

Previous studies have demonstrated that medium-chain triglycerides can significantly improve cognitive function in patients with early-to-intermediate stage AD [207] whereas brain and circulating cholesterol levels are inversely related with motor dysfunction in PD patients [208]. In patients with high cholesterol levels, some studies suggest an elevated PD risk [209]. Finally, the potential associations between ApoB and both ALS and AD risk were observed [205]. However, more studies are necessary in order to clarify the relationship between the heterogeneity of the lipid profiles in the different neurodegenerative diseases.

Conflicting information also emerged after our review regarding the effects of statin therapy on the natural history of the discussed diseases, according to several studies. For ALS, no correlation between statin use and the incidence of ALS was shown. More promising evidence emerged for statin use and AD e PD. However, additional large-scale prospective randomized control studies are needed to better clarify this issue.

## Data Availability

The original contributions presented in this study are included in the article. Further inquiries can be directed to the corresponding author.

## Abbreviations

Central nervous system	CNS
Genome-wide association studies	GWAS
Neurodegenerative diseases	NDDs
Alzheimer’s disease	AD
Parkinson’s disease	PD
Extracellular vesicles	EVs
Amyotrophic lateral sclerosis	ALS
Low density lipoprotein-cholesterol	LDL-C
Peripheral nervous system	PNS
Lipid-lowering molecules	LLMs
Amyloid-β	Aβ
Sphingolipids	SPs
Triglycerides	TGs
High density lipoprotein–cholesterol	HDL-C
Pittsburgh Compound B	PIB
Phospholipid transporter	PLTP
Neurofibrillary tangle	NFTs
Blood–brain barrier	BBB
Multiple sclerosis	MS
Body mass index	BMI
Glucocerebrosidase	GBA
